# Redo isolated tricuspid valve replacement in a patient with isolated persistent left superior vena cava: a case report

**DOI:** 10.1186/s44215-024-00160-8

**Published:** 2024-08-21

**Authors:** Ryotaro Yamada, Homare Okamura, Rie Iwasaki, Atsushi Yamaguchi

**Affiliations:** Department of Cardiovascular Surgery, Saitama Medical Center, Jichi Medical University, 1-847 Amanumacho, Omiya-ku, Saitama, 330-0834 Japan

**Keywords:** Redo isolated tricuspid valve replacement, Structural valve deterioration, Isolated persistent left superior vena cava

## Abstract

**Background:**

Redo isolated tricuspid valve surgery has high in-hospital mortality and morbidity and is a challenging procedure. We report a successful case of redo isolated tricuspid valve replacement for structural valve deterioration of a bioprosthesis in a patient with isolated persistent left superior vena cava (PLSVC).

**Case presentation:**

An 81-year-old man with a history of tricuspid valve replacement using a porcine bioprosthetic valve 9 years previously presented with dyspnea on exertion. Right heart failure due to worsening transvalvular leakage in the bioprosthetic tricuspid valve was considered to be the cause of his symptoms, and the decision was made to replace the tricuspid valve. An isolated PLSVC is considered to be an obstacle in right-sided heart valve surgery. The PLSVC was located deep to the left of the pulmonary artery and, after some effort, was cannulated by obtaining an excellent surgical view using retraction sutures on the left side of the pericardium. Cardiopulmonary bypass was initiated after cannulation of the ascending aorta, PLSVC, and femoral vein. After cross-clamping of the ascending aorta, cold blood cardioplegic arrest was induced under moderate hypothermia, and the PLSVC and inferior vena cava were snared. The right atrium was opened and the prosthetic tricuspid valve was examined. One of the leaflets was shortened, which appeared to cause the transvalvular leak. The prosthetic valve was explanted, the annulus was trimmed, and a new bioprosthetic valve was implanted. The postoperative course was uneventful.

**Conclusions:**

It is important to treat structural valve deterioration of a prosthetic tricuspid valve in a timely manner. We hope that our intervention timing and surgical strategy can help surgeons to consider early intervention in similar cases, even if there are surgical obstacles such as isolated PLSVC.

**Supplementary Information:**

The online version contains supplementary material available at 10.1186/s44215-024-00160-8.

## Background

Redo isolated tricuspid valve surgery (TVS) has high in-hospital mortality and morbidity and is a challenging procedure [1]. There are surgical difficulties such as dissecting adhesion around an expanded and fragile right atrium, superior vena cava (SVC), and inferior vena cava (IVC), which have a high risk of injury. Also, determining the optimal intervention timing to structural valve deterioration (SVD) is difficult, and delayed intervention results in poor outcomes [2].

During embryonic development, left superior vena cava (LSVC) usually regresses, leaving only right superior vena cava (RSVC). However, in 0.2–3% of individuals, LSVC persists. Since persistent left superior vena cava (PLSVC) drains into the coronary sinus (CS), increased venous return results in a dilated CS. RSVC also exists in 90% of patients with LSVC, indicating that isolated PLSVC without RSVC is relatively rare [3]. We report a successful case of redo isolated tricuspid valve replacement (TVR) for SVD of a bioprosthesis in a patient with isolated PLSVC.

## Case presentation

**Fig. 1:**
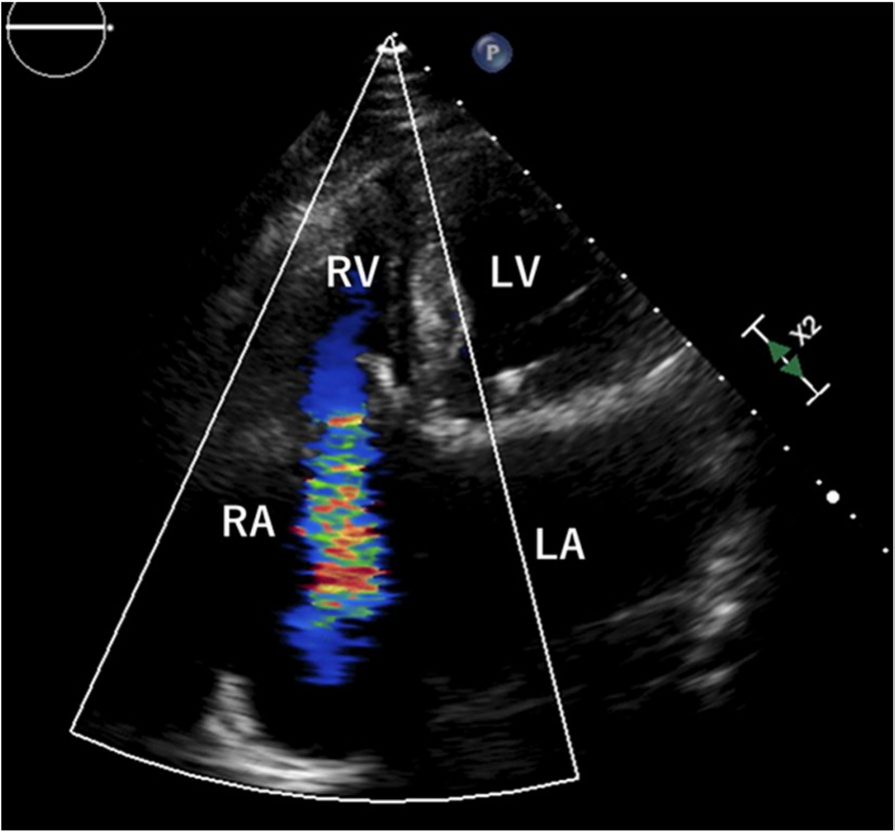
Preoperative transthoracic echocardiography shows severe transvalvular leakage after tricuspid valve replacement. *LA* left atrium, *LV* left ventricle, *RA* right atrium, *RV* right ventricle

**Fig. 2:**
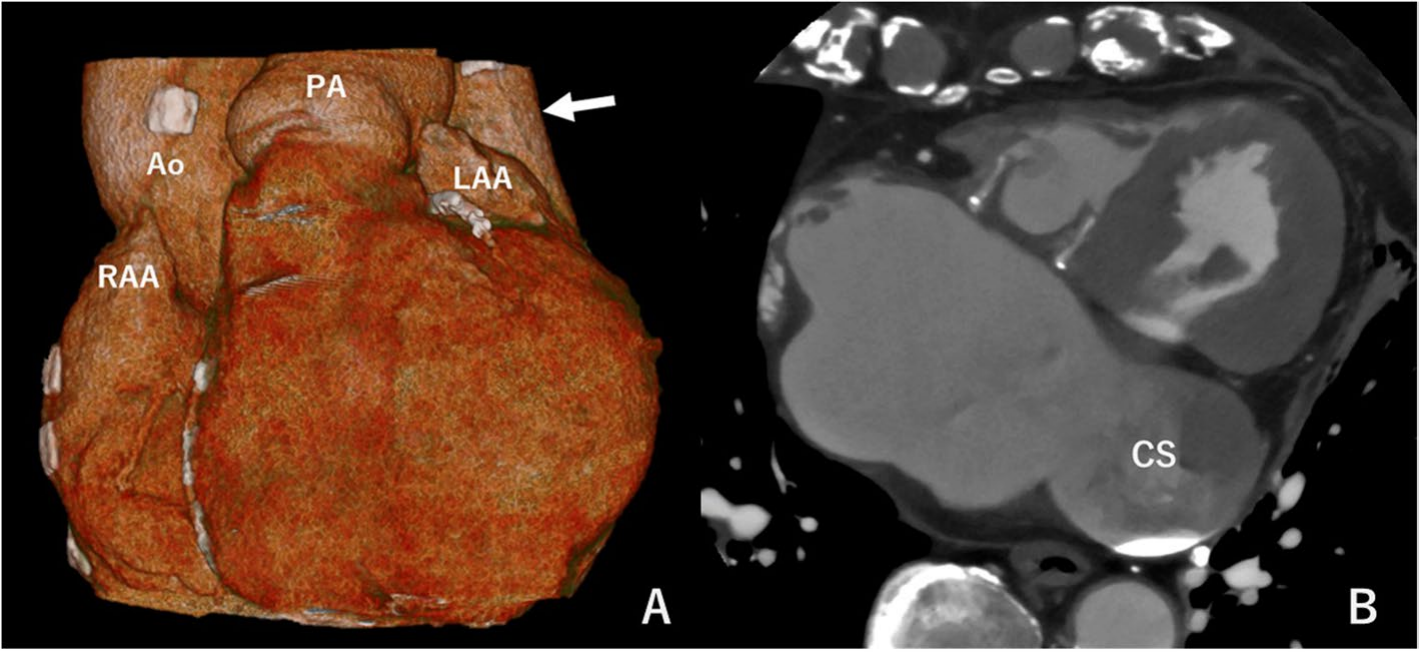
**A** Three-dimensional computed tomography image shows persistent left superior vena cava (white arrow). **B** Axial image shows enlarged coronary sinus (maximum diameter, 42 mm). *Ao* aorta, *CS* coronary sinus, *LAA* left atrial appendage, *PA* pulmonary artery, *RAA* right atrial appendage

An 81-year-old man underwent mitral and tricuspid valve replacement using an epic bioprosthetic valve (Abbott, USA) for severe mitral and tricuspid valve regurgitation. TVR was performed because the tricuspid valvular annulus was too large to control regurgitation by annuloplasty. He also had a history of epicardial permanent pacemaker implantation on the right ventricle for atrial fibrillation with bradycardia after the surgery. Eight years later, the patient presented with dyspnea on exertion. Diuretics that the internist had prescribed was effective, and the symptom improved immediately. However, 1 year later, the symptom reoccurred and worsened despite taking diuretics. Transthoracic echocardiography (TTE) revealed severe transvalvular leakage (TVL) in the tricuspid position (Fig. 1). Right ventricle function before the previous surgery was moderate with tricuspid annular plane systolic excursion (TAPSE) of 14 mm, and it had not changed visually 9 years after surgery. We concluded that redo TVR before deterioration of right cardiac function would be a better option than medical management. Computed tomography revealed a PLSVC draining into the CS (Fig. 2A, B).

**Fig. 3:**
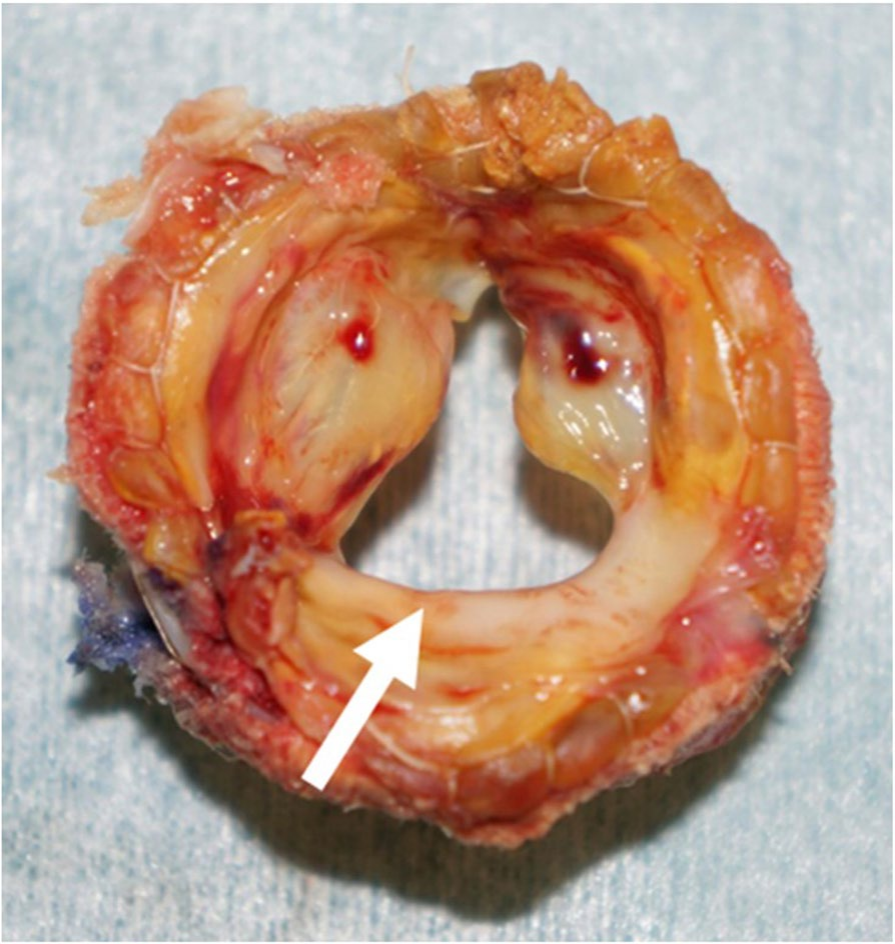
Intraoperative image of the explanted bioprosthesis shows a shortened leaflet without mobility (white arrow)

Median resternotomy was performed without injury to the substernal tissues. The PLSVC was located deep to the left of the pulmonary artery and, after some effort, was cannulated by obtaining an excellent surgical view using retraction sutures on the left side of the pericardium. An additional movie file shows this in more detail (see Additional file 1). CPB was initiated by cannulating the ascending aorta, PLSVC, and femoral vein. A left ventricular vent was inserted through the right upper pulmonary vein. IVC was dilated owing to high venous pressure and safely encircled under decompression. After cross-clamping of the ascending aorta, cold blood cardioplegic arrest was induced under moderate hypothermia, and the PLSVC and IVC were snared. The right atrium was opened. One of the leaflets of the bioprosthetic tricuspid valve was shortened without mobility, causing severe TVL (Fig. 3). The impaired bioprosthesis was removed. A mitral bioprosthetic valve (Edwards Lifesciences, USA) was implanted in the supra-annular position using 2–0 polyester sutures with pledgets. Weaning from CPB was uneventful. The patient was extubated on postoperative day 1. Postoperative TTE revealed no transvalvular or paravalvular leakage in the tricuspid position. The postoperative course was uneventful, with relief of symptoms, and the patient was discharged on postoperative day 14. Three months later, dyspnea on exertion had improved.

## Discussion and conclusions

Redo isolated TVS is typically associated with high in-hospital mortality and morbidity. It has an even higher mortality rate of 12.3% in Japan, which is much higher than that of aortic or mitral valve surgery [1]. The most important reason for high in-hospital mortality in redo isolated TVS is missing the appropriate timing for the procedure [2]. Major indications for redo isolated TVS are prosthetic valve endocarditis and heart failure due to SVD. Timing of surgery is often delayed because it is difficult to choose surgery during the early stage of heart failure when symptoms quickly improve with the use of diuretics. After stress from continuous severe tricuspid valve regurgitation has been placed on the right heart, refractory heart failure—which is resistant to useful drugs—eventually occurs. In fact, some studies have suggested that New York Heart Association (NYHA) function class IV is an independent risk factor for in-hospital deaths and that the main cause of in-hospital deaths is multiorgan failure due to end-stage heart failure [2]. Thus, early intervention is recommended to achieve better outcomes.

There were also surgical difficulties associated with our case. Both inserting venous cannulas into PLSVC and IVC and snaring them are necessary to maintain bloodless surgical view inside the right-side of the heart. Careful handling is necessary when dissecting around the PLSVC and IVC. The PLSVC was located deep and far from the midline. Pulling the left side of the pericardium anteriorly was helpful to obtain exposure of the PLSVC. If we could not insert a venous cannula into the PLSVC, venous cannulation of the CS during CPB was an option [4]. However, in our case, it was a challenging option because the CS was too large (maximum diameter, 42 mm) to snare it completely with a purse-string suture (Fig. 2B). The right atrium and IVC were dilated due to a long period of tricuspid valve regurgitation. The risk of IVC injury is high; thus, a venous cannula was inserted via the femoral vein. The reason is that, even if taping and snaring the IVC cannot be performed, clamping the IVC is possible to provide a bloodless surgical view of the right heart. Although, we fortunately were able to snare both the PLSVC and IVC after some effort, these surgical plans were important to prevent in-hospital mortality following redo isolated TVS.

In conclusion, it is important to ensure appropriate timing of surgical intervention for SVD of a tricuspid prosthetic valve. We hope that our intervention timing and surgical strategy can help surgeons to consider early intervention in similar cases, even if there are surgical obstacles such as isolated PLSVC.

## Supplementary Information

Additional file 1. Intraoperation video. Dissecting adhesion around the PLSVC.

## Data Availability

Not applicable.

## Abbreviations

TVS: Tricuspid valve surgery
SVC: Superior vena cava
IVC: Inferior vena cava
TVR: Tricuspid valve replacement
SVD: Structural valve deterioration
PLSVC: Persistent left superior vena cava
RSVC: Right superior vena cava
CS: Coronary sinus
TTE: Transthoracic echocardiography
TVL: Transvalvular leakage
TAPSE: Tricuspid annular plane systolic excursion
CPB: Cardiopulmonary bypass
NYHA: New York Heart Association
